# Supplementary material for: Characterization of Gene Expression Suppression by Bovine Coronavirus Non-Structural Protein 1
Source: Viruses. 2025 Jul 13;17(7):978. doi: 10.3390/v17070978 (PMC12299145; doi:10.3390/v17070978)
Supplement: Supplementary file 1 [file viruses-17-00978-s001.zip › viruses-3664889-supplementary.pdf]

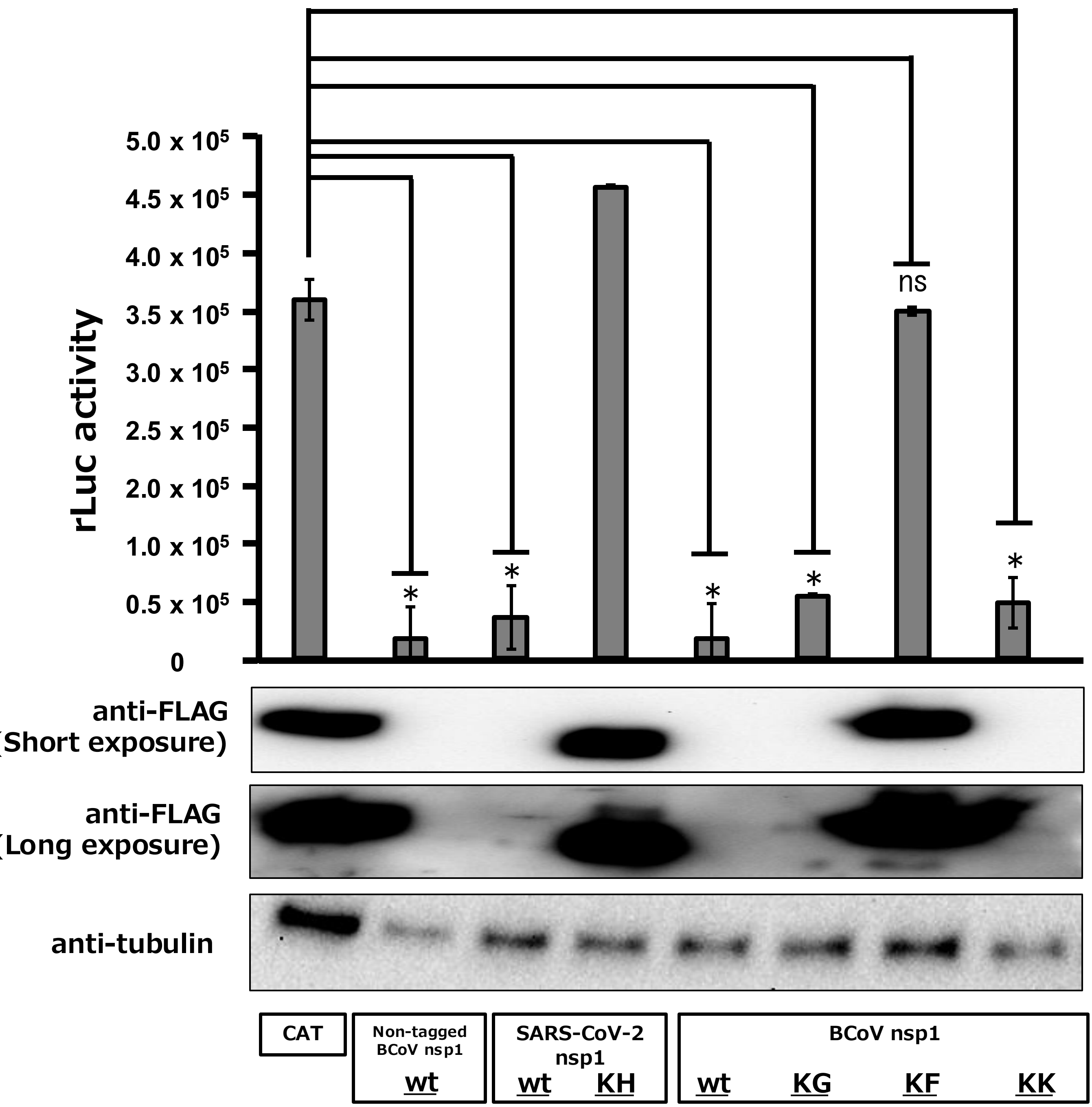

Supplemental Figure S1. Results of reporter assay in MAC-T cells by using N-terminal FALG-tagged nsp1s. We cotransfected MAC-T cells with pRL-TK (encoding the rLuc gene) and pCAGGS-BCoV nsp1-wt (encoding BCoV nsp1), pCAGGS-BCoV nsp1-KF (encoding BCoV nsp1-KF), pCAGGS-BCoV nsp1-KG (encoding BCoV nsp1-KG), or pCAGGS-BCoV nsp1-KK (encoding BCoV nsp1-KK). As a control, pCAGGS-CAT (encoding the CAT gene), pCAGGS- SARS-CoV-2 nsp1-wt (encoding SARS-CoV-2 nsp1), or pCAGGS-SARS-CoV-2 nsp1-KH mt (encoding biologically inactive SARS-CoV-2 nsp1-mt) was used in place of pCAGGS-BCoV nsp1-wt and -mts. Expressed CAT and nsp1s, but not non-tagged BCoV nsp1, carried the N-terminal FLAG tag. (A) At 24 h post-transfection, cell lysates were prepared and subjected to luciferase assay. Error bars show the standard deviations of results from three independent experiments. Asterisks represent significant differences in rLuc activity ($P < 0.01$). ns, not significant. Western blot analysis of the cell extracts was performed to detect plasmid-expressing proteins (B) and tubulin (C)..

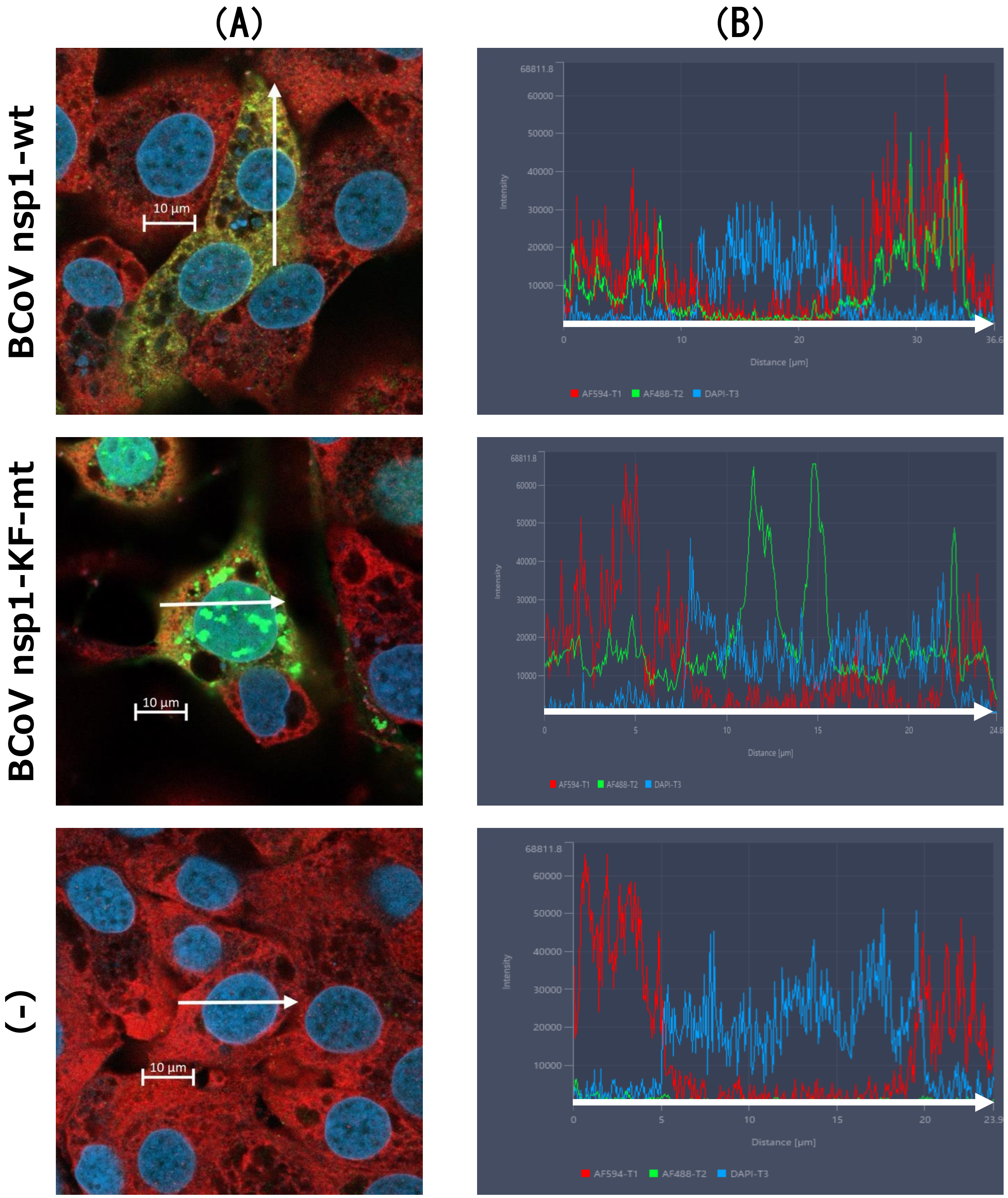

Supplemental Figure S2. Representative confocal micrographs of MAC-T cells expressing BCoV nsp1-wt or BCoV nsp1-KF MAC-T cells were mock transfected or transfected with pCAGGS-BCoV nsp1-wt (encoding C-terminal FLAG-tagged BCoV nsp1) or pCAGGS-BCoV nsp1-KF (encoding C-terminal FLAG-tagged BCoV nsp1-KF). At 24 h post-transfection, the cells were fixed, permeabilized, and subjected to immunofluorescence analysis with an anti-FLAG antibody and anti-S6 ribosomal protein. The nuclei were counterstained with Hoechst 33342, and the images were examined using a Zeiss LSM900 confocal microscope. Scale bar, 10 mm. The right panels show the intensity profile analysis generated using Zen Software to display the distribution of the red (S6 ribosomal protein), green (BCoV nsp1-wt or KF), and blue (nuclei) fluorophore signals.

```
MERS-CoV    --------------------------------MSFVAGVTAQGARGTYRAALNSEKHQDH    28
SARS-CoV2   --------------------------------------------MESLVPGF---NEKTH    13
MHV         MAKMGKYGLGFKWAPEFPWMLPNASEKLGNPERSEEDGFCPSAAQEPKVKGK---TLVNH    57
BCoV        MSKINKYGLELHWAPEFPWMFEDAEEKLDNPSSSEVDIVCSTTAQKLETGGI---CPENH    57
HCoVOC43    MSKINKYGLELHWAPEFPWMFEDAEEKLDNPSSSEVDMICSTTAQKLETDGI---CPENH    57
                                                              .        *

MERS-CoV    VSLTVPL------CGSGNLVEKLSPWFMDGENA-YEVVKAM----LLKKEPLL-YVPIRL    76
SARS-CoV2   VQLSLPV----------LQVR---DVLVRGFGDSVEEVLSEARQHLKDGTCGLVE-----    55
MHV         VRVNCSRLPALECCVQSAIIR---DIFVDEDPQKVEASTMMA---LQFGSAVLVKPSKRL    111
BCoV        VMVDCRRLLKQECCVQSSLIR---EIVMNTRPYDLEVLLQDA---LQSREAVLVTPPLGM    111
HCoVOC43    VMVDCRRLLKQECCVQSSLIR---EIVMNASPYYLEVLLQDA---LQSREAVLVTTPLGM    111
            * :                :.     .:       *         *      *

MERS-CoV    A----GHTRHLP-GPRVYLVERLIAC-------ENPFMVNQLAYSSSANGSLVGTTLQGK    124
SARS-CoV2   -----VEKGVLPQLEQPYVFIKRSDA---RTAPHGHVMVE--LV-AELEGIQYG--RSGE    102
MHV         SIQAWTNLGVLPKTAAMGLFKRVCLCNTRECSCDAHVAFH--LFTVQPDGVCLG---NGR    166
BCoV        SLEACYVRGCNPNGWTMGLFRRRSVCNTGRCAVNKHVAYQ--LYMIDPAGVCFG---AGQ    166
HCoVOC43    SLEACYVRGCNPKGWTMGLFRRRSVCNTGRCTVNKHVAYQ--LYMIDPTGVCLG---AGQ    166
                       *      :. :   .       .  .  .      .  *   *    *.

MERS-CoV    PIGMFFPYDIEL------VTGKQNILLRKYGRGGYHYTPFHYE--------RDNTSCPEW    170
SARS-CoV2   TLGVLVPHVGEIPVAY------RKVLLRKNGNKGAGGHS--YGAD-LKSFDLGDELGTDP    153
MHV         FIGWFVPV-TAIPEYAKQWLQPWSILLRKGGNKGSVTSGHFRRAVTMPVYDFNV---EDA    222
BCoV        FVGWVIPL-AFMPVQSRKFIVPWVMYLRKCGEKGAYNKDHKRG-GFEHVYNFKV---EDA    221
HCoVOC43    FVGWVIPL-AFMPVQSRKFIVPWVMYLRKRGEKGAYNKDHGCG-GFGHVYDFKV---EDA    221
             :* ..*    :            : *** *. *                        :

MERS-CoV    MDDFEADPKGKYAQNL----LKKLIGGDV    195
SARS-CoV2   YEDFQENWNTKHSSGVTRELMRELNGG--    180
MHV         CEEVHLNPKGKYSC-KAYALLKGYRG---    247
BCoV        YDLVHDEPKGKFSK-KAYALIRGYRGV--    247
HCoVOC43    YDQVHDEPKGKFSK-KAYALIRGYRGV--    247
             : .. : : *.:       ::   *
```

Supplemental Figure S3. Alignment of the amino acid sequences of nsp1s of MERS-CoV, SARS-CoV-2, MHV, human coronavirus OC43, and BCoV . Nsp1 sequences of the MERS-CoV strain EMC2012 (accession no.: YP_009047229), SARS-CoV-2 isolate Wuhan-Hu-1 (accession no.: MN908947.3), MHV (accession no.: AF029248.1), human coronavirus OC43 (accession no.: MN306036), and BCoV (accession no.: LC642814.1) are aligned using Multiple Sequence Comparison by Log-Expectation, alignment algorithm. Perfect matches, high-amino acid similarities, and low-amino acid similarities are represented by asterisks, double dots, and single dots, respectively. A dash "-" indicates a gap in the sequence. The numbers beside the aligned sequences show the positions of amino acid residues. The residues shown in red represent the functional amino acids for host translation inhibition. The residues shown in green represent the functional amino acids for host mRNA cleavage. Residues shown in blue represent the amino acids that are important for virulence of MHV and mouse-adopted SARS-CoV.

Supplemental Table S1 Primers used for plasmid construction

Plasmid	Amino Acid Mutation	PCR	Forward or Reverse	Sequence (5'-3')	Amplification Size
pCAGGS-BCoV nsp1-KG	KG-to-AA	1st PCR ①	Forward	AAGAATTCGCCACCATGTCGAAGATCAACAAATACGGT	725 bp
			Reverse	AGCCTTCTTAGAAAACTTAGCAGCAGGCTCATCATGAACCAA	
		1st PCR ②	Forward	TTGGTTCATGATGAGCCTGCTGCTAAGTTTTCTAAGAAGGCT	369 bp
			Reverse	TATAGCCACCTTTGTTCATGGCAG	
		2nd PCR	Forward	AAGAATTCGCCACCATGTCGAAGATCAACAAATACGGT	1052 bp
			Reverse	TATAGCCACCTTTGTTCATGGCAG	
pCAGGS-BCoV nsp1-KF	KF-to-AA	1st PCR ①	Forward	AAGAATTCGCCACCATGTCGAAGATCAACAAATACGGT	725 bp
			Reverse	AGCATAAGCCTTCTTAGAAGCAGCACCCTTAGGCTCATCATG	
		1st PCR ②	Forward	CATGATGAGCCTAAGGGTGCTGCTTCTAAGAAGGCTTATG	369 bp
			Reverse	TATAGCCACCTTTGTTCATGGCAG	
		2nd PCR	Forward	AAGAATTCGCCACCATGTCGAAGATCAACAAATACGGT	1052 bp
			Reverse	TATAGCCACCTTTGTTCATGGCAG	
pCAGGS-BCoV nsp1-KK	KK-to-AA	1st PCR ①	Forward	AAGAATTCGCCACCATGTCGAAGATCAACAAATACGGT	725 bp
			Reverse	TCTAATTAAAGCATAAGCAGCAGCAGAAAACTTACCCTTAGG	
		1st PCR ②	Forward	CCTAAGGGTAAGTTTTCTGCTGCTGCTTATGCTTTAATTAGA	369 bp
			Reverse	TATAGCCACCTTTGTTCATGGCAG	
		2nd PCR	Forward	AAGAATTCGCCACCATGTCGAAGATCAACAAATACGGT	1052 bp
			Reverse	TATAGCCACCTTTGTTCATGGCAG	
pCAGGS-BCoV nsp1	(-)	1st PCR	Forward	AAGAATTCGCCACCATGTCGAAGATCAACAAATACGGT	1052 bp
			Reverse	TATAGCCACCTTTGTTCATGGCAG	

Supplemental Table S2 Primers to amplify housekeeping genes for the standard curve

Target gene	Forward or Reverse	Sequence (5'-3')	Length of PCR product
GAPDH	Forward	CATCACCATCTTCCAGGAGC	546 bp
	Reverse	CTGGAGAAACCTGCCAAGTA	
HPRT1	Forward	GAGATGTGATGAAGGAGATGGGTGG	689 bp
	Reverse	CTGCATTCCCGAACTCTTCATTTGC	
18s ribosomal RNA	Forward	ACTGAGGCCATGATTAAGAGG	586 bp
	Reverse	CCACCCGAGATTGAGCAATA	
